# Effect of N-Acetyl Cysteine on Intracerebroventricular Colchicine Induced Cognitive Deficits, Beta Amyloid Pathology, and Glial Cells

**DOI:** 10.1155/2019/7547382

**Published:** 2019-04-15

**Authors:** Teresa Joy, Muddanna S. Rao, Sampath Madhyastha, Keshav Pai

**Affiliations:** ^1^Department of Anatomy, Kasturba Medical College, Mangalore, Manipal Academy of Higher Education, India; ^2^Department of Anatomy, Faculty of Medicine, Kuwait University, Kuwait; ^3^Department of Psychiatry, Kasturba Medical College, Mangalore, Manipal Academy of Higher Education, India

## Abstract

Among the many factors responsible for the cognitive decline in Alzheimer's disease, beta amyloid protein and plaque formation is crucial. This amyloid pathology is associated with activation of glial cells and oxidative stress but whether oxidative stress activates beta amyloid protein in the neurons is not clear. Further the expression of microglia is also known to vary during pathogenesis of beta amyloid plaques. The aim of the present study is to evaluate the antioxidant effect of NAC on amyloid pathology and cognition and also to investigate the link between amyloid pathology and glial cells activation. Intracerebroventricular colchicine in rats known mimics human AD in many aspects including memory loss, oxidative stress, and hyper phosphorylation of tau protein. The animal groups consisted of age matched control, sham operated, AD, and NAC treated in AD models of rats. Cognitive function was evaluated in active avoidance test; beta amyloid protein, beta amyloid plaques, astrocytes, and microglia cells were quantified using immunohistochemistry in hippocampal and prefrontal cortices. Colchicine has resulted in significant cognitive loss, increased intraneuronal beta amyloid protein expression, increased reactive astrocytes, and activated microglia in all the regions of the hippocampus and prefrontal cortices. The antioxidant NAC has reversed the cognitive deficits and inhibited microglia activation but failed to inhibit BAP expression and astrocytosis. Intraneuronal BAP accumulation is deleterious and known to adversely affect cognition, but in this study in spite of intraneuronal BAP accumulation, the cognition is restored. It can be postulated that NAC might have reversed the effect of intraneuronal beta amyloid protein by acting on some downstream compensatory mechanisms which needs to be explored.

## 1. Introduction

Alzheimer's disease (AD) is a nonreversible, progressive, devastating neurodegenerative disease characterized by memory loss which is associated with neuronal loss. Its pathological features are abnormal buildup of extracellular amyloid plaques [1], intracellular neurofibrillary tangles [2], cholinergic deficiency [3], loss of synaptic connections [4], and its subsequent consequence the inhibition of neuronal signaling and neuronal loss. The inhibition of neuronal signaling in the hippocampal network is the major cause for memory loss and cognitive impairment in AD. Beta amyloid protein (BAP) is a derivative from a glycoprotein named amyloid precursor protein. Damage to neurons causes accumulation of BAP, which is due to consecutive cleavage of the APP in the cell membrane. Then by series of reaction (*β*-secretase and  *γ*-secretase cleaves APP) BAP is released to the extracellular space [5]. The disparity between BAP creation and BAP clearance is the root cause for the creation of amyloid plaques. It was believed that BAP is synthesized only in neurons; however, recent finding indicates that astrocytes play a further role in AD by synthesizing significant amounts of BAP [6, 7]. Since astrocytes are numerous in the brain, even minor quantity of amyloid secretion from astrocytes could be substantial. AD is often characterized by increase in reactive astrocytes close to the sites of amyloid plaques [8]. 

Oxidative stress occurs during progression of AD in presence of BAP. Elevated levels of BAP are consisted of increased levels of oxidation products in hippocampus and cortex [9] of AD patients. In addition to this, the cellular stressor can increase APP expression and therefore increase BAP secretion [10]. It is believed that the oxidative stress on the action of *γ*-secretase (enzyme which cleaves APP) in astrocytes is the primary cause for production of BAP [11] apart from other conditions. Hence boosting the antioxidant defense in astrocytes and neurons would minimize the BAP, *β*-amyloid plaques, and memory loss.

Whether BAP accumulation is a cause or consequence of AD remains a question. Studies targeting BAP with several *γ*-secretase inhibitors, which have efficiently reduced BAP levels, have been unsuccessful in clinical trials. Further, *γ*-secretase inhibitors are known to have adverse effects on cognition [12]. This low efficacy of present therapeutics for the treatment of AD has guided the researchers to look at alternate avenues. BAP accumulation is mainly initiated and enhanced by oxidative stress [13]. Several antioxidants have been testified to inhibit the formation of *β*-amyloid plaques or BAP and also destabilize them. In studies in transgenic mice model, some antioxidant compounds reduced plaque load* in vivo* [14]. Glutathione (GSH) and thioredoxin are two intracellular antioxidants in addition to other antioxidants obtained from diet help in normalizing the ageing induced alteration [15, 16]. It has been revealed that the level of GSH is reduced in hippocampus and cortical areas of patients with AD as compared with controls [17]. N-acetyl cysteine (NAC) is a derivative of amino acid, cysteine, and a precursor in the formation of the antioxidant glutathione in the body. NAC's neuroprotective action is through restoration of glutathione pool [18] and direct scavenging ability against reactive species [19]. However little attention has been focused on the effect of NAC on BAP pathology except for a preclinical study that provided some evidence that administration of NAC is beneficial in transgenic mouse model of AD by decreasing BAP [20].

Microglial cells generation is triggered in presence of *β*-amyloid plaques in neocortex [21]. There is another study suggesting that beta amyloid plaques cause loss of microglial cells and inhibition of neural stem cells [22]. BAP cause oxidative stress through microglial activation [23]. Oxidative stress and neuroinflammation together create a malicious cycle in AD pathology [24]. Microglial activation and oxidative stress can be increased as a result of BAP formation. Therefore, mediations that reduce BAP-induced microglial activation and oxidative stress might be useful for AD treatment. If an antioxidant treatment prevents plaque formation, thereby reducing the microglial expression or enhancing the reactive microglial expression for phagocytosis of these plaques is not clear. Hence the present study would provide facts about the expression of microglial cells in an animal model of AD.

Although no single animal model recapitulates all of the features of the AD, each model allows for in-depth analysis of only one or two components of the disease. Colchicine, a microtubule distracting agent, causes damage of neurons through neurofibrillary disintegration [25], BAP expression [26], oxidative stress [27], and neuronal loss; all these features closely simulates human AD [28]. Moreover, the present study is aimed at looking at the effect of glutathione supplement on amyloid pathology. Hence in this experiment of an* in vivo *model of AD, we test the ability of NAC in minimizing BAP, *β*-amyloid plaques, cognitive loss, and also its effect on expression of astrocytes and microglia.

## 2. Methods

### 2.1. Animals

In-house bred male albino* Wistar *rats, four months old and weighing 250-270g, were used in this study. Rats were fed with water and food* ad libitum*. The rats were maintained under controlled conditions of light-dark cycle (12:12), temperature (22±3°C), humidity (50±10%), and pathogen-free environment. Polypropylene cage with paddy husk as bedding material was used for housing the rats. The experimental procedure was approved by Institution Animal Ethics Committee (IAEC/KMC/2012).

### 2.2. Animal Groups

The rats were randomly divided into the following five groups (n=12 in each group). i) Control- rats in this group remained in the home cage without any surgical procedure and were treated with saline throughout the experimental period (2 weeks). ii) Sham-rats in this group underwent a sham surgical procedure, where skull surface was exposed, a bur hole was drilled aiming to the lateral ventricle, a 32G needle was lowered into lateral ventricle, 5*μ*l of sterile artificial CSF was injected and needle was withdrawn, and finally skin was sutured. These rats were treated with saline throughout the experimental period. iii) Alzheimer's disease- (AD-) rats in this group were injected with colchicine into ventricle stereotaxically (15*μ*g) to induce Alzheimer's disease. These rats were treated with saline throughout the experiment. Iv) Alzheimer's treated with 50mg/kg of NAC- (AD+NAC-50-) rats in this group were injected with colchicine into ventricle stereotaxically to induce Alzheimer's like disease and were treated with NAC (50mg/kg, i.p.) throughout the experiment. V) Alzheimer's treated with 100mg/kg of NAC- (AD+NAC-100-) rats in this group were injected with colchicine into ventricle stereotaxically to induce Alzheimer's-like disease, and were treated with NAC (100mg/kg, i.p.) throughout the experiment. Rats in all groups were subjected to active avoidance learning and memory test after treatment period (2 weeks)

### 2.3. Chemicals

NAC was purchased from Lobo Chemicals (Mumbai, India). Artificial cerebrospinal fluid (ACSF: in m mol/l:147NaCl, 2.9 KCl, 1.6 MgCl_2_, 1.7 CaCl_2_ and 2.2 dextrose) was obtained from Biotech India Pvt. Ltd. (New Delhi, India). Colchicine was obtained from Sigma Aldrich (Sigma chemicals, St. Louis, MO, USA). Rabbit polyclonal anti-beta amyloid antibody (Cat#-ab2539) known to express neuronal cytoplasm as beta amyloid protein (BAP) and extra neuronal beta amyloid plaques was obtained from abcam (Cambridge, MA, USA), rabbit polyclonal antiglial fibrillary acidic protein (GFAP) for astrocytes was from Dako Flex (Cat#-IS524, Agilent Technologies India Pvt. Ltd., Bangalore, India), and rabbit monoclonal anti-Iba1 for microglia was from abcam (Cat#-ab178847, Cambridge, MA, USA). All other chemicals and reagents are HPLC or analytical grade were from Sigma Aldrich (Sigma chemicals, St. Louis, MO, USA).

### 2.4. Surgery and Intracerebroventricular (ICV) Administration of Colchicine

To create an Alzheimer's-like model, colchicine (a microtubule disrupting agent, also known to cause oxidative stress) was injected into the lateral ventricle (either left or right) stereotaxically. Stereotaxic surgical procedure was as described in our previous study [29]. Briefly, the rats were anesthetized with sodium pentobarbital (40mg/kg, i.p.) and skull was exposed with a midline skin incision. A bur hole was drilled on the skull cap at the following stereotaxic coordinate: Anteroposterior, 0.8mm behind the bregma, and lateral, 2mm from midline [30, 31]. The skull cap was drilled carefully up to the level of dura mater, without damaging any nervous tissue. A 32G needle connected to one end of a capillary tube was held in the needle holder of the stereotaxic apparatus and inserted through the bur hole to a depth of 3.2mm from skull surface aiming at the lateral ventricle. Other end of the capillary tube was connected to a Hamilton microsyringe filled with colchicine (or artificial cerebrospinal fluid for sham group). Hamilton microsyringe was positioned in an infusion pump (Harvard apparatus). 5*μ*l of artificial cerebrospinal fluid or 15*μ*g colchicine in 5*μ*l of artificial cerebrospinal fluid was injected slowly over a period of 20 minutes. Needle was held in place for an additional 5 minutes before withdrawal, in order to prevent the backflow of the injected materials. Thereafter the needle was gently removed, and the scalp was closed with sutures. Antibiotics were applied on the surgical wound to prevent any infection. The rats were kept in a warm place until they recover from anesthesia. Special care was taken during the postoperative period to provide food and water inside the cage of the rat. Following surgery, the rats were housed individually in cages until end of the experiment.

#### 2.4.1. NAC Administration

NAC in physiological saline was administered intraperitoneally, one week prior to surgery and one week following the surgery at 50mg/kg or 100mg/kg dose. The doses of NAC were selected based on earlier studies [32], and human dose calculated for rats. Twelve rats were used for cognitive test in each group. Out of the twelve rats six were randomly selected for immunohistochemical studies.

### 2.5. Active Avoidance Test

This test was employed to evaluate associative learning and memory retention, at the end of treatment period. In this test, ability of the rat to evade an aversive experience by learning to accomplish a specific behavior in response to a stimulus signal is assessed. The shuttle box apparatus used for this test was a closed wooden box with shutter doors in the front wall. The floor area consisted of a stainless-steel metal grid, separated into two compartments by a median wall with an open door interconnecting two compartments. The floor grid of the two compartments was connected to an electric stimulator. A buzzer was installed inside shuttle box to give a discriminative sound stimulus during behavioral test. Behavioral test consisted of i) exploration, ii) active avoidance learning, and iii) memory retention test. Exploration was on 1^st^ day of test, where rats were placed in the box and allowed to explore both compartments of the apparatus for 5min to make them familiar with shuttle box. Active avoidance learning test was for five consecutive days. Rat was placed in one of the compartments and allowed to explore both compartments for five minutes. At the end of exploration, a discriminative sound stimulus was provided through the buzzer, during which the rat could move to other compartment in order to avoid the foot shock. If the rat failed to move to other compartment, as soon as it hears the discriminative sound stimulus, a foot shock (2.5mA) was delivered through the grid floor for a maximum of 10 seconds, during which it could cross to the other compartment and escape the foot shock. The possibility for avoiding the foot shock was a single crossing over to other compartment of the shuttle box. The active avoidance learning consisted of 30 trials/day for 5 consecutive days. The ‘number of shock avoidances/day' (total number of shock avoidances in 5 days divided by 5) was calculated for each rat. In the initial trials, rats fail to associate the sound stimulus to shock avoidance, but in the subsequent trails they learned to associate sound stimulus to shock avoidance by actively moving to the other compartment. In the control rats, the number of shock avoidances increases from day 1 to 5 of the test. Any decrease in the number of shock avoidances/day is an indication of learning impairment. Memory retention test was done one week after the last learning trial to assess memory retention. Memory retention test was similar to the learning trails but for only one day. A comparison of rat's performance in learning trails with its performance during memory retention gives the assessment of memory and is presented as the percentage of memory retention score (% RTS), which was calculated by using the following formula [29]. Any decrease in the % RTS and number of shock avoidances/day during retention test is an indication of memory impairment.

(1) $$\begin{array}{c} \%\;\;RTS=\frac{No.\;\;of\;\;shock\;\;avoidance/day\;\left(retention\;\;test\right)\times No.\;\;of\;\;shock\;\;avoidance/day\;\left(learning\;\;trails\right)}{Number\;\;of\;\;shock\;\;avoidance\;\;on\;\;5th\;\;day\;\;of\;\;learning.}\times 100 \end{array}$$

### 2.6. Tissue Processing for Immunostaining

A day after the active avoidance test, six rats from each group were deeply anesthetized with ether and perfused with saline followed by freshly prepared 4% paraformaldehyde in phosphate buffer (pH=7.4). The brains were postfixed in 4% paraformaldehyde in phosphate buffer for 48 hours. Brain tissues were processed for paraffin blocks. Coronal sections (7*μ*) from prefrontal cortex and hippocampus region were cut using a rotary microtome (Jung Biocutt 2035, Lieca, Wetztar, Germany, [30]. Five sections from each rat brain were mounted on gelatin coated slides and air dried.

### 2.7. Immunostaining for BAP, *β*-amyloid Plaques, Astrocytes, and Microglia

The paraffin sections were immunostained with anti-beta amyloid antibody for expression BAP in the neurons and beta amyloid plaques outside the neurons [33], antiglial fibrillary acidic protein (GFAP) for astrocytes [34], and anti-Iba1 for microglia in the prefrontal cortical regions and hippocampal subregions. Sections were deparaffinized in xylene and rehydrated in descending grades of ethyl alcohol. Antigen retrieval was done by incubating the sections in 0.1M citrate buffer at 60°C for 30 min. The sections were treated with 3% H_2_O_2_ for 30 minutes to reduce the endogenous peroxidase activity in the tissue. Sections were then incubated for 30 minutes with 5% normal goat serum along with 0.3% Triton X-100 in PBS (pH7.4) to block the nonspecific binding of the primary antibody. The sections were incubated with rabbit polyclonal anti-beta amyloid antibody (1:500) or rabbit polyclonal antiglial fibrillary acidic protein (GFAP, 1:500) or rabbit monoclonal anti-Iba1 (1:1000) overnight at 4°C. The sections were incubated with biotinylated goat-anti-rabbit IgG as secondary antibody (1: 200, Vector Laboratories, Burlingame, CA) for 1hr at room temperature. The sections were washed in PBS and treated with avidin-biotin-peroxidase complex (ABC kit, Vector Laboratories, Burlingame, CA) for 1hr at room temperature. Subsequently color was developed with 3,3′-diaminobenzidine as chromogen (DAB, Vector Laboratories, Burlingame, CA). Throughout the staining protocol, sections were washed three times with PBS after each incubation. To assess nonspecific staining, several sections in each experiment were incubated in buffer without primary antibody. Sections were lightly counterstained with hematoxylin, dehydrated in ascending ethanol grades, cleared in xylene, and cover slipped in Permount (Fisher Scientific, Pittsburgh, PA).

### 2.8. Quantification of BAP Positive Neurons, *β*-amyloid Plaques, Astrocytes, and Microglia

High quality images were captured with 40x objective, with an Olympus digital camera (DP75) attached to an Olympus microscope. In each image immunostained BAP positive neurons or GFAP positive astrocytes or Iba1 positive microglia were counted using NIS Elements Br version 4.30 software. In each hippocampal section, 300 *μ*m length of Cornu Ammonis subregions (CA1, CA2, CA3, and CA4) and 300 *μ*^2^ area of the dentate gyrus (DG) were selected for quantification. In prefrontal cortex, the number of neurons/astrocytes/microglia in 300 *μ*^2^ area was counted in medial, lateral, and orbital regions of the prefrontal cortex. Slides from different groups of rats were coded to avoid manual bias while counting the cells.

### 2.9. Statistical Analysis

The data were expressed as mean ± SE and were analyzed with one-way ANOVA, followed by Bonferroni's multiple comparison post hoc test useing SPSS (version 25) statistical analysis software. P values <0.05 were considered as significant. The treatment effect between 2 doses of NAC was assessed through paired Student's t-test.

## 3. Results

### 3.1. Memory Retention Test

**Figure 1:**
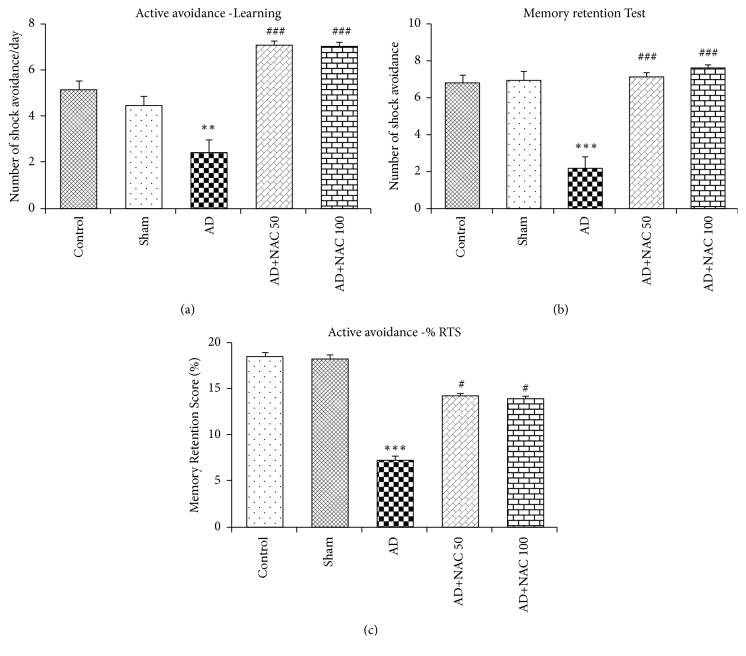
Performance of rats in different groups in active avoidance learning (a) and in memory retention test ((b) and (c)). Note that AD rats had significant learning and memory deficit and deficits were decreased/normalized by treatment with NAC (both 50mg/kg and 100mg/kg). Control/sham versus AD: ∗∗∗, p<0.001; AD versus AD+NAC50 or AD versus AD+NAC100: ###, p<0.001 (One-way ANOVA, Bonferroni's multiple comparison test, n=6 in all groups).

The mean number of shock avoidance/day (mean of 5 days' avoidance), mean number of shock avoidance during retention test and % retention score (%RTS) did not differ between control and sham operated rat groups. ICV colchicine (AD like disease in rats) had significantly reduced mean number of avoidance/day during learning (p<0.01), during memory retention test (p<0.001) and % retention score (p<0.001), clearly demonstrating learning disability and memory impairment (Figures 1(a)–1(c)). NAC treatment (at both the doses) in AD model of rats has significantly increased mean number of avoidance/day during learning (Figure 1(a), p<0.001), during memory retention test (p<0.001, Figure 1(b)) and % retention score (p<0.05, Figure 1(c)) compared to rats receiving only colchicine. The number of shock avoidances/day during learning and during memory retention test and % retention score (%RTS) did not differ between control rats and AD + NAC group of rats. There was no significant (p>0.05) dose-dependent effect on any of the parameters studied. This is an indication that IVC colchicine induced cognitive loss and poor memory retention was almost reversed and brought back to the control levels (Figures 1(a)–1(c)).

### 3.2. BAP Positive Neurons and *β*-amyloid Plaque Expression

**Figure 2:**
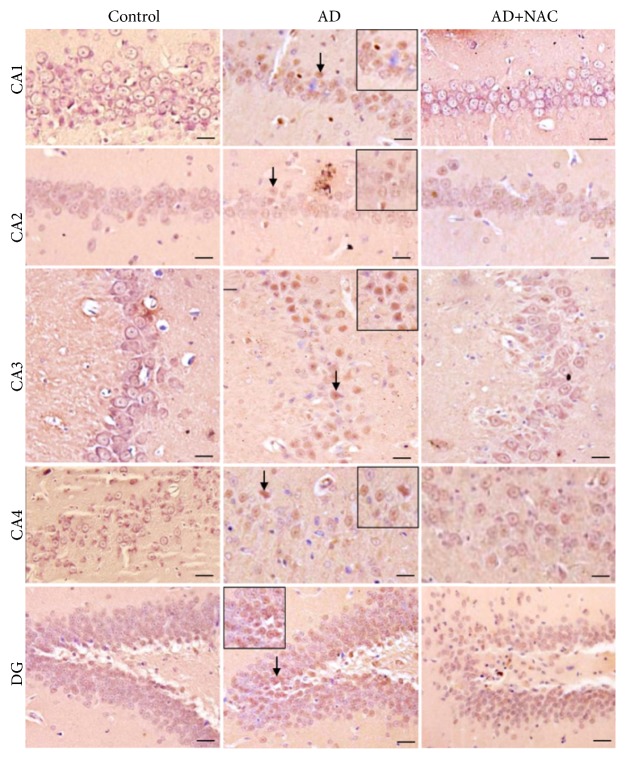
Photomicrographs of the hippocampal subregions in different groups of rats immunostained for *β*-amyloid protein (BAP). Note the expression of BAP (arrow) in large number of neurons in AD group in all regions. The number of BAP positive neurons was less or not present in NAC treated groups (AD+NAC 100) (photomicrographs of sham and AD+NAC 50 groups were avoided for simplicity). Scale bar= 25*μ*, in CA1-CA4 regions, =15*μ* in dentate gyrus (DG).

**Figure 3:**
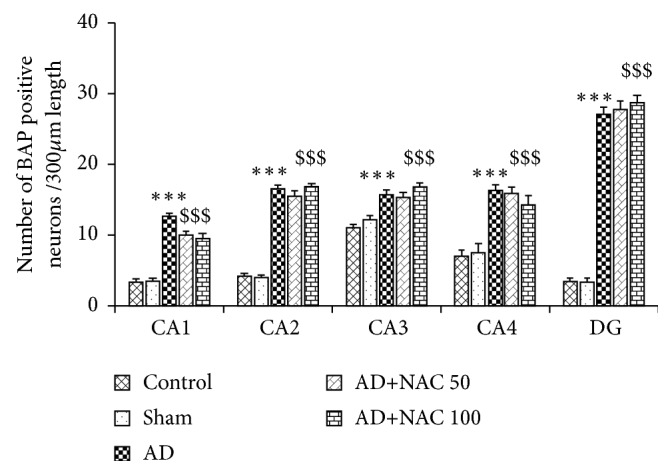
Quantitative estimation of number of neurons expressing BAP in various regions of the hippocampus. In CA1, CA2, CA3, and CA4 regions 350 *μ*m length and in dentate gyrus (DG) 150*μ*^2^ area were selected for quantification. Note that, in all regions, number of neurons expressing BAP significantly increased in AD and AD+NAC 50, AD+NAC 100 groups compared to control group. Values are expressed as mean ± SE. Control/sham versus AD: ∗∗∗, p<0.001; control/sham versus AD+NAC50 or control/sham versus AD+NAC100: $$$, p<0.001 (one-way ANOVA, Bonferroni's multiple comparison test, and n=6 in all groups).

**Figure 4:**
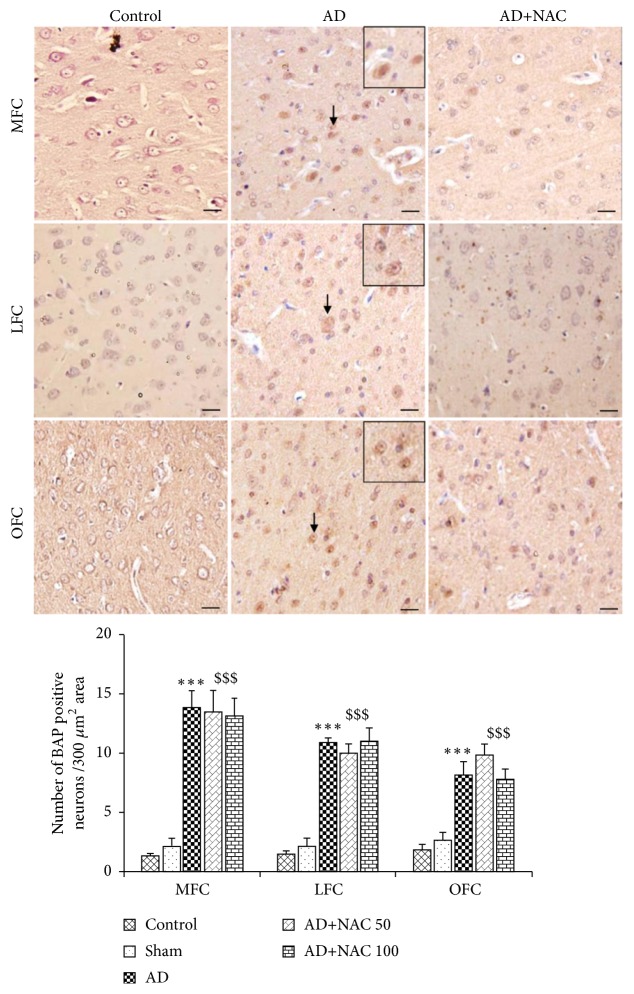
Photomicrographs of the prefrontal cortical regions in different groups of rats immunostained for *β*-amyloid protein (BAP). Note the expression of BAP (Arrow) in large number of neurons in AD group in all regions. The number of BAP positive neurons was less or not present in NAC treated groups (AD+NAC 100) (photomicrographs of sham and AD+NAC 50 groups are avoided for simplicity). Scale bar= 25*μ*. Graph shows quantitative estimation of number of neurons expressing BAP in frontal cortical regions. In all regions 300*μ*^2^ area was selected for quantification. Note that, in all regions, number of neurons expressing BAP significantly increased in AD and AD+ NAC 50, AD+NAC 100 groups compared to control group. Values are expressed as mean ± SE. Control/sham versus AD: ∗∗∗, p<0.001; control/sham versus AD+NAC50; or control/sham versus AD+NAC100: $$$, p<0.001 (one-way ANOVA, Bonferroni's multiple comparison test, and n=6 in all groups). MFC-Media prefrontal cortex, OFC-Orbital prefrontal cortex, and LFC-Lateral prefrontal cortex.

Amyloid plaques were sparsely distributed in the AD model of rats. They were of diffuse variety and very rarely compact or dense-cored plaques were seen. Positive intracellular staining for BAP was observed in AD model of rats. Amyloid deposits in blood vessels were abundant in many areas of the brain investigated including hippocampal subregions (Figure 2). Cannula implantation has not significantly (p>0.05) affected the total number of BAP positive neurons compared to control rats in any regions of the hippocampus or prefrontal cortices. The number of BAP positive cells were significantly (p<0.001) high in all the regions of the hippocampus in AD model of rats compared to control or sham operated group of rats (Figure 3). Neurons in the prefrontal cortex also expressed *β*-amyloids extensively and the number of BAP positive cells was significantly high in AD model of rats compared to control or sham operated group of rats (p<0.001, Figure 4). NAC treatment at 50 or 100mg/kg dose showed a significant increase in BAP positive cells compared to control or sham operated groups (p<0.001, Figures 3 and 4). Further there was no significant difference in number of BAP positive cells expression between AD group of rats compared to AD rats who received NAC in all the regions of the hippocampus except in CA1 region. Similar results were observed in prefrontal cortices also (Figure 4). In CA1 region, the BAP positive cells were significantly more in AD rats treated with NAC compared to AD rats.

### 3.3. GFAP Expression

**Figure 5:**
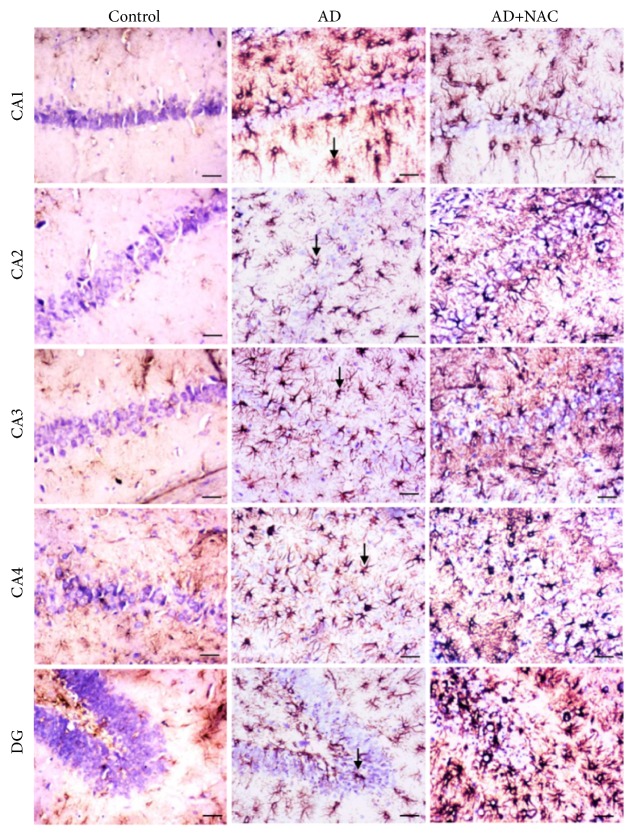
Photomicrographs of the hippocampal subregions in different groups of rats immunostained for glial acidic protein (GFAP). Note the expression of GFAP (arrow) in large number in AD and AD+NAC group in all regions. The number of GFAP positive astrocytes was more in AD and AD+NAC treated groups (AD+NAC 100) (photomicrographs of sham and AD+NAC 50 groups are avoided for simplicity). Scale bar= 25*μ*, in CA1-CA4 regions, =15*μ* in dentate gyrus (DG).

**Figure 6:**
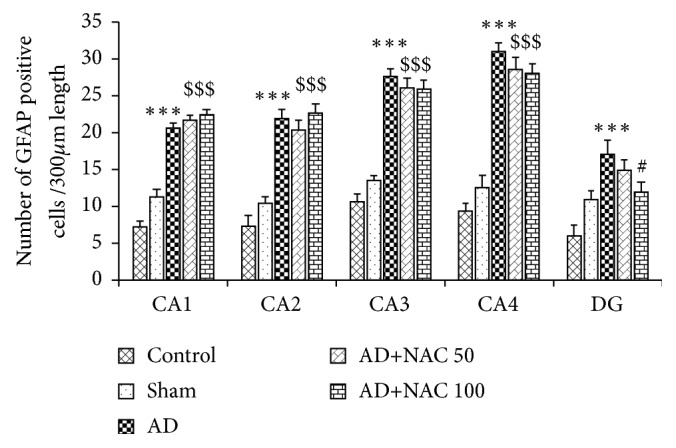
Quantitative estimation of number of GFAP positive astrocytes in various regions of the hippocampus. In CA1, CA2, CA3, and CA4 regions 350 *μ*m length and in dentate gyrus (DG) 150*μ*2 area were selected for quantification. Note that, in all regions, the number of astrocytes was significantly increased in AD and AD+ NAC 50, AD+NAC 100 groups compared to control group. In dentate gyrus, number of astrocytes in AD+NAC-100 significantly decreased compared to AD group. Values are expressed as mean ±SE. Control/sham versus AD: ∗∗∗, p<0.001; control/sham versus AD+NAC50; or control/sham versus AD+NAC100: $$$, p<0.001, AD versus AD+NAC 100: #, p< 0.05 (one-way ANOVA, Bonferroni's multiple comparison test, and n=6 in all groups).

Distribution of GFAP positive astrocytes in the hippocampal subregions and in the dentate gyrus is shown in Figure 5. The density of reactive astrocytes was higher in AD and AD+ NAC group of rats in all regions compared to control rats. The reactive astrocytes were expressed abundantly at the area surrounding the neurons of hippocampus and also adjacent areas. In AD model of rats and also AD model who received NAC, the astrocytes were expressed with sharp dendritic margins which are not observed in control rats. Cannula implantation has not significantly (p>0.05) affected the astrocytes expression compared to control rats in any regions of the hippocampus or prefrontal cortices. The number of GFAP positive cells were significantly high in all the hippocampal subregions in AD models of rats compared to their control counterpart (p<0.001, Figure 6). Further NAC treatment (both the doses) in AD rats did not show any significant difference in GFAP expression compared to AD models of rats in the hippocampal subregion.

**Figure 7:**
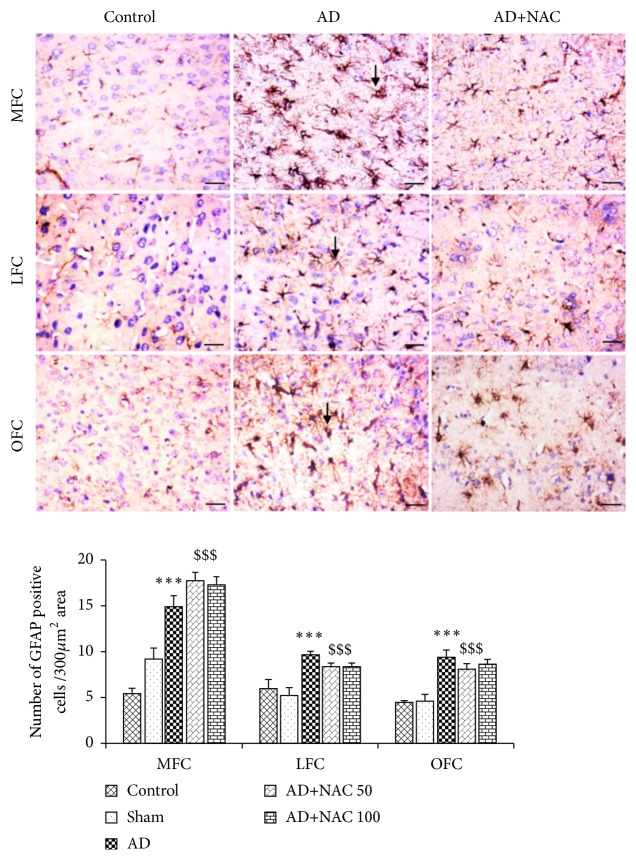
Photomicrographs of the prefrontal cortical regions in different groups of rats immunostained for glial acidic protein (GFAP) to label the astrocytes. Note large number of astrocytes in AD and AD+NAC group in all regions. The number of GFAP positive astrocytes was more in AD and AD+NAC treated groups (AD+NAC 100) (photomicrographs of sham and AD+NAC 50 groups are avoided for simplicity). Scale bar= 25*μ*). Graph shows quantitative estimation of number of GFAP positive astrocytes in various regions of the prefrontal cortex. In all regions 300*μ*2 area was selected for quantification. Note that, in all regions, the number of astrocytes was significantly increased in AD and AD+ NAC 50, AD+NAC 100 groups compared to control group. Values are expressed as mean ±SE. Control/sham versus AD: ∗∗∗, p<0.001; control/sham versus AD+NAC50; or control/sham versus AD+NAC100: $$$, p<0.001 (one-way ANOVA, Bonferroni's multiple comparison test, and n=6 in all groups). MFC-Media prefrontal cortex, OFC-Orbital prefrontal cortex, and LFC-Lateral prefrontal cortex.

The pattern of distribution of GFAP positive astrocytes in the prefrontal cortical regions is shown in Figure 7. As in the hippocampus and dentate gyrus, the astrocytes are densely packed in AD and AD+ NAC groups compared to control rats. GFAP positive cells in AD and AD+NAC were significantly (p<0.001) high in all the three prefrontal cortices examined compared to their control counterparts (Figure 7). However, sham operated group did not show any significant increase in GFAP expression compared to control. NAC at both doses did not alter GFAP expression compared to AD model of rats. It was also observed that the GFAP expression in NAC treated AD models of rats was significantly high compared to control group of rats (p<0.001, Figure 7).

### 3.4. Expression of Iba1 Positive Microglia

**Figure 8:**
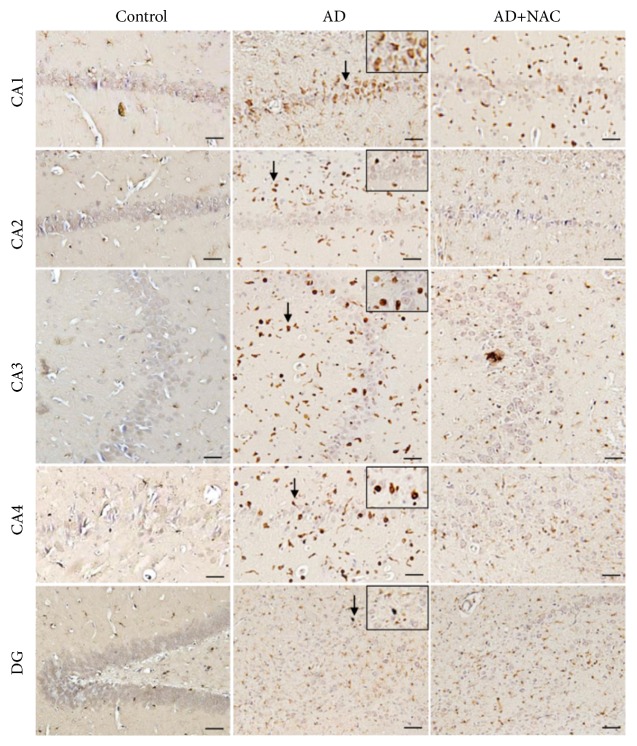
Photomicrographs of the hippocampal subregions in different groups of rats immunostained for Iba1 to label reactive microglia. Note the presence of reactive microglia (Arrow) in large number in AD group in all regions and their distribution is less dense in AD+NAC group (photomicrographs of sham and AD+NAC 50 groups are avoided for simplicity). Scale bar= 25*μ*, in CA1-CA4 regions, =15*μ* in dentate gyrus (DG).

**Figure 9:**
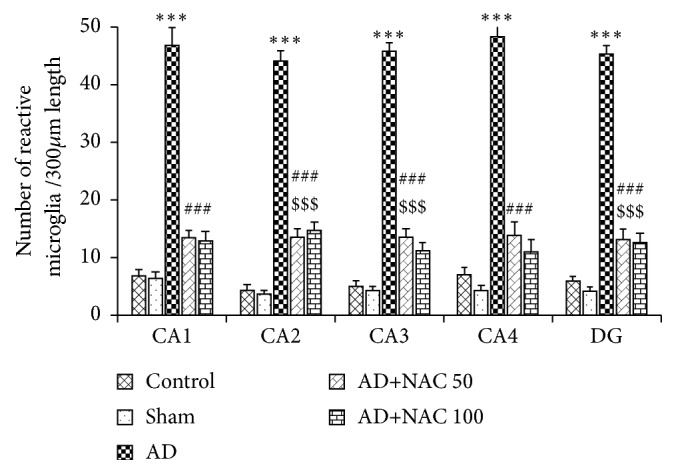
Quantitative estimation of number of Iba1 positive reactive microglia in various regions of the hippocampus. In CA1, CA2, CA3, and CA4 regions 400*μ*m length and in dentate gyrus (DG) 400*μ*2 area were selected for quantification. Note that, in all regions, the number of microglia was significantly increased in AD group compared to control group. Number of microglia decreased significantly in AD+NAC 50 and AD+NAC 100 compared to AD group. In CA2, CA3, and DG number of microglia significantly increased compared to control group. Values are expressed as mean ± SE. Control/sham versus AD: ∗∗∗, p<0.001; control/sham versus AD+NAC50; or control/sham versus AD+NAC100: $$$, p<0.001, AD versus AD+NAC 50/100: ###, p< 0.001 (one-way ANOVA, Bonferroni's multiple comparison test, n=6 in all groups).

**Figure 10:**
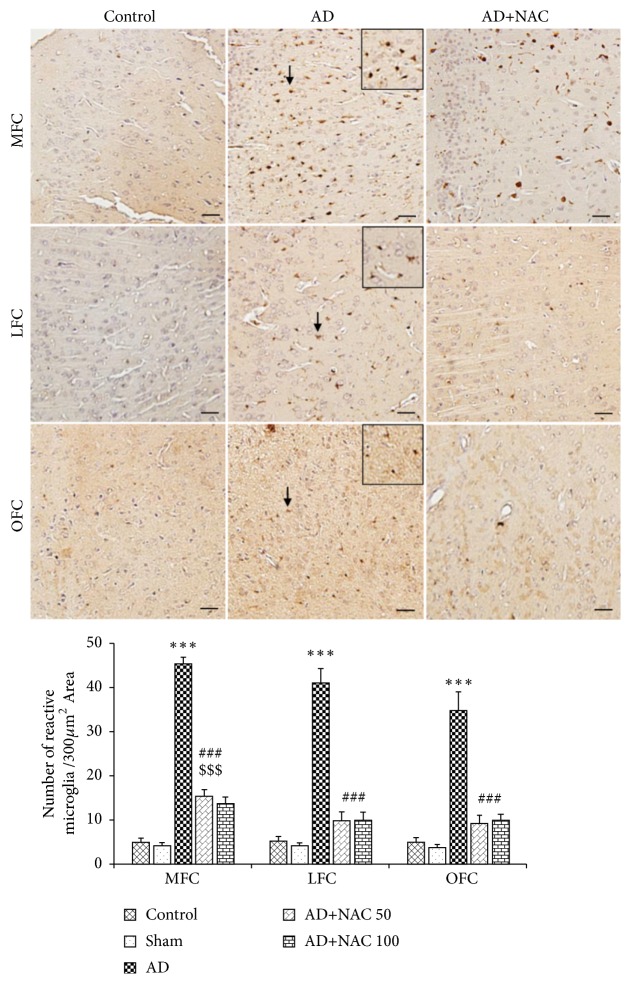
Photomicrographs of the frontal cortical regions in different groups of rats immunostained for Iba1 to label reactive microglia. Note the presence of reactive microglia (arrow) in large number in AD group in all regions and their distribution is less dense in AD+NAC group (photomicrographs of sham and AD+NAC 50 groups are avoided for simplicity). Scale bar= 25*μ*. Graph shows quantitative estimation of number of Iba1 positive reactive microglia in various prefrontal cortical regions. In all regions 400*μ*2 area was selected for quantification. Note that, in all regions, the number of microglia was significantly increased in AD group compared to control group. Number of microglia decreased significantly in AD+NAC-50 and AD+NAC 100 compared to AD group. In MCF, number of microglia significantly increased compared to control group. Values are expressed as mean ±SE. Control/sham versus AD: ∗∗∗, p<0.001; control/sham versus AD+NAC50; or control/sham versus AD+NAC100: $$$, p<0.001, AD versus AD+NAC 50/100: ###, p< 0.001 (one-way ANOVA, Bonferroni's multiple comparison test, and n=6 in all groups). MFC-Media prefrontal cortex, OFC-Orbital prefrontal cortex, and LFC-Lateral prefrontal cortex.

The Iba1 positive microglia were found abundantly at the area away from the neurons of hippocampus and prefrontal cortex unlike astrocytes expression which are more closely associated with neurons (Figures 8 and 10). Cannula implantation has not significantly (p>0.05) affected the microglia expression compared to control rats in any regions of the hippocampus or prefrontal cortices. The number of activated microglia was significantly high in AD model of rats compared to control or sham operated group in all the regions of the hippocampus (p<0.001, Figure 9). This indicates that ICV colchicine induces activation of microglia. NAC treatment at both doses in AD rats has significantly reduced the expression of activated microglia compared to AD model of rats in all the regions of the hippocampus (p<0.001, Figure 9). This is an indication that NAC has exerted its neuroprotective effect by minimizing the gliosis. The quantitative data on activated microglia in AD model of rats treated with either 50 or 100 mg/kg dose of NAC did not show any statistically significant (p>0.05) difference with control group of rats in CA1 and CA4 regions. However, in CA2, CA3, and DG regions, the AD model of rats treated with NAC (either 50 or 100mg/kg dose) expressed a significantly (p<0.001) higher number of reactive microglia compared to control rats. This can be assumed that the NAC has exerted its restored effect in CA1 and CA4 regions compared to the remaining subregions of the hippocampus. The expression of activated microglia was significantly more in AD model of rats compared to control or sham operated rats in all the three regions of the prefrontal cortex investigated (p<0.001, Figure 10). This indicates that ICV colchicine has caused microgliosis in prefrontal cortices. NAC treatment (either 50 or 100 mg/kg dose) in AD model of rats significantly reduced the expression of activated microglia in all the three prefrontal cortices compared to AD model of rats (p<0.001, Figure 10). This indicates the neuroprotective effect of NAC against colchicine induced microgliosis. Further in the comparison between AD rats treated with NAC and control rats, the MFC regions showed a significant difference but not in the LFC and OFC (p<0.001, Figure 10). This indicates that NAC has exerted its protective effect better in LFC and OFC compared to MFC.

## 4. Discussion

The major findings of this experiment are that a week after ICV colchicine administration to rats resulted in severe cognitive deficit. Immunohistochemistry conducted in these rats after cognitive test (about 26 days after colchicine administration) revealed a significantly higher number of BAP positive neurons in the hippocampus and prefrontal cortices, the areas concerned with cognition. Further this is accompanied with gliosis involving both astrocytes and microglia in hippocampus and prefrontal cortices. The cognitive deficits observed a week after colchicine administration involves amyloid pathology and gliosis in hippocampus and prefrontal cortices. The upregulated intraneuronal BAP expression, which is more toxic than plaques, and the neuroinflammation observed by gliosis is the factors responsible for cognitive impairment. NAC treatment a week before and a week after colchicine administration has reversed the cognitive deficits. However, NAC treatment did not alter the intraneuronal BAP expression and also the expression of reactive astrocytes in hippocampus and prefrontal cortices. However, the activated microglia expression was downregulated after NAC administration. The reversal of cognitive deficits after NAC treatment in AD model of rats is associated with reduction in microglia expression but not BAP positive neurons or expression of reactive astrocytes.

Many transgenic AD animal models which expressed accumulation of BAP inside and outside the neurons showed severe cognitive deficits [35]. In our study, the colchicine treated AD model of rats exhibited similar intraneuronal BAP in cortical and hippocampal areas which is associated with cognitive impairment. ICV colchicine induced oxidative stress and concomitant increase in BAP level of hippocampal tissue was demonstrated before [36, 37]. ICV colchicine is also known to cause excessive free radical generation and oxidative damage [38]. Oxidative stress induced BAP accumulation and resultant cognitive loss were reported earlier [39, 40]. Hence cognitive deficits in the form of disability in learning and memory observed in this study can be positively correlated to BAP and probably the oxidative stress, though we did not measure the oxidants in the brain. In this study, enhancing the brain antioxidant capacity by NAC treatment was able to reverse the cognitive deficits. It is well-known that oxidative stress is responsible for changes in the neurons and behavioral deficits in AD [41]. In previous studies, NAC has reversed behavioral deficits observed in traumatic brain injury in several animal models [42–44] due its antioxidant potential. NAC treatment in mice receiving ICV injections of BAP had improved learning and memory compared to vehicle-treated animals [45]. This study claims that the cognitive deficits observed is due to upregulation of BAP expression and this part of their findings was similar to our findings in this study. However, unlike their study, we noticed that NAC treatment has reversed the cognitive deficits in spite of higher level of BAP expression in hippocampal and prefrontal cortices. This is an interesting finding but it requires further studies to evaluate this phenomenon. We can only assume that the choice of behavioral study model used in our study evaluates a form of procedural memory which is likely to be affected only in late stages of AD. It can also be postulated that NAC might have reversed the effect of intraneuronal beta amyloid protein by acting on some downstream compensatory mechanism which needs to be explored. NAC may have a role in disintegration of BAP and beta amyloid plaques. Production of BAP within the neurons results from two proteases cleaving APP: *β*-secretase and *γ*-secretase. NAC is known to inhibit APP gene transcription [46]. It has significantly decreased soluble levels of BAP in transgenic mice that overexpress the APP gene [20]. There are many studies that demonstrate that NAC is capable of curbing amyloid pathology in AD model of rats. However, our study did not demonstrate any noticeable effect of NAC in amyloid pathology. Hence the beneficial effect of NAC observed in reversing the cognitive deficits involves higher complex mechanisms and least likely due to amyloid pathology alone.

The neuroglial interactions are key in neurotransmission and any interruption in glial functions also contribute to cognitive dysfunctions. Microglia and astrocytes are activated in regions of the brain occupied by amyloid plaques and also oxygen free radicals [47, 48] which are characteristics of AD. In AD, there is an increase in the number of activated microglia and reactive astrocyte close to the sites of amyloid plaques [8]. These reactive astrocytes surrounding beta amyloid plaques are the cause for local inflammatory response and it modifies calcium signaling [49, 50]. Loss of astroglial function and reactivity contribute to neurodegenerative diseases like AD [51]. In this study we observed activation of astrocytes and higher number of reactive microglia expression (microgliosis) in almost all the areas of hippocampus and prefrontal cortices in presence of BAP positive neurons. Transcription of proinflammatory cytokines and chemokines occur due to BAP-induced intracellular signaling pathways. This can result in cellular damage to the astrocytes or even stimulate BAP in astrocytes [52]. Astrocytes appear to be the chief target of BAP, as this protein brings several effects of oxidative stress including defective intracellular calcium signaling [53]. Though we could not demonstrate BAP in astrocytes or altered calcium signaling, activation of astrocytes was very much evident in all the areas we investigated. Apart from this, the gliosis observed in this study could be due to colchicine induced neuroinflammation and neuronal damage or oxidative stress.

Adequate evidence suggests that amyloid plaques are not randomly distributed in the brain but show a characteristic spatial pattern. Studies showed that CA1 is one of the most affected regions in AD, mainly at early stages. In our study ICV colchicine has resulted in BAP expression in all the subregions of the hippocampus which also showed severe gliosis. In addition to that in CA1 region the number of BAP positive cells was more in AD model of rats who received NAC compared to rats who received only colchicine.

In our previous study we demonstrated that ICV colchicine causes upregulation of hyper phosphorylated tau protein with considerable neuronal loss and cognitive decline. In the same experiment NAC administration has reversed tau pathology, cognitive deficits, and also the neuronal loss [54]. Correlating with earlier findings, it can be postulated that NAC can reverse tau pathology but not amyloid pathology. The reversal of cognitive function may be due to downregulation of tau protein expression in hippocampus and prefrontal cortices and also reduced expression of activated microglia. It is largely believed that the tau aggregation is probably induced by b-amyloids and neurofibrillary tangles appear in the brain later than senile plaques. However, it has also been suggested that accumulation of A*β* plaques does not correlate with cognitive impairments in AD patients. A large number of individuals without any cognitive impairment accumulate A*β* plaques in their brains [55, 56]. Another interesting factor is that *β*-amyloid plaque accumulation is not intrinsically cytotoxic and also that BAP does not induce tau accumulation [57]. In a recent review article by Kametani and Hasegawa [58] they claimed that AD is a disorder that is triggered by impairment of APP metabolism and progresses through tau pathology, not A*β* amyloid.

Astrocytes are involved in maintaining or processing oxidative stress in AD. Astrocytes have a key role in maintaining the neuronal integrity; damaged or activated astrocytes are vulnerable to neuronal functions. Thus activated astrocytes observed in this study might have caused oxidative stress and inhibited axonal transmission which resulted in cognitive dysfunction. It can also be correlated that overexpression of BAP, as observed in this study, has caused oxidative stress in neurons as well as astrocytes. Excessive BAP is known to induce oxidative stress in brain [52]. Astrocytes are the producers of the raw materials needed for the production of glutathione in neurons [59]. It can be assumed that upregulation of BAP in astrocytes could prevent glutathione production. Preclinical data also provide evidence that NAC treatment is beneficial in AD murine models counteracting oxidative damage [60]. Further Tucker and coworkers [20] demonstrated antiamyloid efficacy of NAC. However, in our study supplementation of NAC, the glutathione precursor did not alter the BAP expression. Hence one of the reasons for continued higher expression of BAP is due to continued higher expression of activated astrocytes. The bioavailability of glutathione is poor (low solubility and absorption, together with a rapid metabolism and elimination); hence testing with higher dose of NAC and measuring glutathione content from hippocampus would likely provide better understanding.

Microglial cells phagocytose beta amyloid plaques, as they express beta amyloid plaque degrading enzymes. They also get activated to produce inflammatory chemokines, cytokines, and neurotoxins [61]. It is suggested that microglial cells play a duel role in the pathogenesis of AD [62]. They are able to clear soluble fibrillar A*β*; however their constant interactions with A*β* can cause an inflammatory response thereby resulting in neurotoxicity. In this study we observed microglial activation in hippocampus and prefrontal cortices. NAC is known to exert its neuroprotective potential through two well-known mechanisms, that is, restoration of glutathione pool [63] and direct scavenging ability against reactive oxygen species [64]. Activation of microglia is a hallmark of neuroinflammation, which enhances the production and release of reactive oxygen species. NAC, the antioxidant, is involved in detoxification of reactive oxygen species in the brain. Hence it is supposed that NAC would inhibit microglial activation. Accordingly, in our study, NAC has inhibited microglial activation in hippocampus and prefrontal cortices in presence of BAP positive neurons. This suggests that oxidative stress induced by colchicine is responsible for microglial activation. BAP initiates a cascade of events, including activation of microglial cells and oxidative stress [65]. But in our study the activation of microglia was inhibited by NAC even in presence of BAP positive neurons. In a rat model of spinal cord injury both astrocytes and microglial expressions were increased [66]. NAC treatment in these rats had no effect on reactive astrocytes but the microglial reaction was significantly decreased. From these findings it can be suggested that NAC has a significantly positive effect on microglia but not on astrocytes. This is possible because the astrocytes itself might have expressed abundant BAP, since NAC has not been able to minimize the BAP expression. Further the response of astrocytes and microglia may be different in presence of amyloid pathology. Evaluating the expression of BAP in astrocytes would likely provide further information.

## 5. Conclusion

ICV colchicine causes intraneuronal BAP expressions and cognitive loss which is associated with gliosis. The antioxidant NAC has reversed the cognitive deficits and inhibited microglia activation but failed to inhibit expression of BAP positive neurons and reactive astrocytes in an animal model of AD. It can be postulated that NAC might have reversed the effect of intraneuronal beta amyloid protein by acting on some downstream compensatory mechanism which needs to be explored.

## Data Availability

The data used to support the findings of this study are included within the article.
